# Supplementary material for: Explicit and Intrinsic Intention to Receive COVID-19 Vaccination among Heterosexuals and Sexual Minorities in Taiwan
Source: Int J Environ Res Public Health. 2021 Jul 7;18(14):7260. doi: 10.3390/ijerph18147260 (PMC8307662; doi:10.3390/ijerph18147260)
Supplement: Supplementary file 1 [file ijerph-18-07260-s001.zip › ijerph-1285316-supplementary.pdf]

Supplementary Table S1. Measures used in this study

Measures	Items	Response scale
Explicit intention to get vaccinated against COVID-19	Please rate your current willingness to receive a COVID-19 vaccine:	1 (very low) to 10 (very high)
Intrinsic Intention to Get Vaccinated for COVID-19 (Drivers of COVID-19 Vaccination Acceptance Scale)	1. Vaccination is a very effective way to protect me against COVID-19.	1 = strongly disagree, 2 = disagree, 3 = slightly disagree, 4 = neither disagree nor agree, 5 = slightly agree, 6 = agree, 7 = strongly agree<br>*: reverse-coded
	2. I know very well how vaccination protects me from COVID-19.	
	3. It is important that I get the COVID-19 jab.	
	4. Vaccination greatly reduces my risk of catching COVID-19.	
	5. I understand how the flu jab helps my body fight the COVID-19 virus.	
	6. The COVID-19 jab plays an important role in protecting my life and that of others.	
	7.* I feel under pressure to get the COVID-19 jab.	
	8. The contribution of the COVID-19 jab to my health and well-being is very important.	
	9. I can choose whether to get a COVID-19 jab or not.	
	10.* How the COVID-19 jab works to protect my health is a mystery to me.	
	11.* I get the COVID-19 jab only because I am required to do so.	
	12. Getting the COVID-19 jab has a positive influence on my health.	
Risk perception of COVID-19	Item 1: If you were to develop flu-like symptoms tomorrow, would you worry?	1 = not at all worried, 2 = worried less than normal, 3 = about the same, 4 = worried more than normal, 5 = extremely worried
	Item 2: In the past one week, have you ever worried about catching COVID-	1 = no, never think about it, 2 = think about it but it didn't

	19?	worry me, 3 = worried me a bit, 4 = worried me a lot, 5 = worried about it all the time
	Item 3: Please rate the current level of your worry towards COVID-19:	Score ranged from 1-10 (1 = very mild, 10 = very severe)
	Item 4: How likely do you think it is that you will contract COVID-19 over the next 1 month?	1 = never, 2 = very unlikely, 3 = unlikely, 4 = evens, 5 = likely, 6 = very likely, 7 = certain
	Item 5: What do you think are your chances of getting COVID-19 over the next 1 month compared to others outside your family?	1= not at all, 2 = much less, 3 = less, 4 = evens, 5 = more, 6 = much more, 7 = certain
